# Irreversibility Marangoni Tri-Hybrid Nanoflow Analysis for Thermal Enhancement Applications

**DOI:** 10.3390/nano13030423

**Published:** 2023-01-19

**Authors:** Malik Zaka Ullah

**Affiliations:** Department of Mathematics, Faculty of Science, King Abdulaziz University, Jeddah 21589, Saudi Arabia; zmalek@kau.edu.sa

**Keywords:** ternary hybrid nanofluids (MoS_2_, SiO_2_, MWCNTs), Marangoni convection, irreversibility analysis, thermal radiation, irreversibility analysis, magnetic field, HAM and numerical solution

## Abstract

Increasing heat transfer is an important part of industrial, mechanical, electrical, thermal, and biological sciences. The aim of this study is to increase the thermal competency of a conventional fluid by using a ternary hybrid nanofluid. A magnetic field and thermal radiation are used to further improve the thermal conductivity of the base fluid. Irreversibility is analyzed under the influence of the embedded parameters. The basic equations for the ternary hybrid nanofluids are transformed from Partial Differential Equations (PDEs) to Ordinary Differential Equations (ODEs) using the similarity concept. The Marangoni convection idea is used in the mathematical model for the temperature difference between the two media: the surface and fluid. The achieved results are provided and discussed. The results show that ternary hybrid nanofluids are more suitable as heat-transmitted conductors than conventional fluids.

## 1. Introduction

The emerging innovative energy sources are posing various problems in the quest to meet the growing demand for energy in advanced scientific and engineering processes. The thermal performance of the fluids used for heat transfer, for the most part, is low, which seriously limits device performance by slowing the performance of the equipment and the compression of the heat exchangers. Choi [1] introduced nanoparticles for thermal and cooling applications. With advances in new procedures, Novoselov et al. [2] applied graphene in thermal and cooling devices. The stable diffusion of these small solid materials in a base liquid produces nanofluids. Improvements in heat transfer mainly depend on (i) the stability of chemical processing, (ii) thermophysical properties, (iii) stable diffusion in the base solvent, (iv) availability, (v) cost, and (vi) toxicity. Materials from the carbon family are regularly used in thermal applications owing to their availability and reasonable cost. These are available in the form of graphene sheets, tubes, oxides, and graphite.

The stability of nanoparticles in a base solvent has been analyzed [3]. The movement of basic liquids was observed by Thomson [4] through experimental analysis. Gul and Firdous [5] experimentally observed the stable diffusion of graphene oxide in water and investigated the quantity of GOW nanofluid amongst two gyrating discs. Over time, researchers have introduced new ideas for the preparation of nanofluids, such as mixing different nanoparticles in the same base liquid to prepare hybrid nanofluids (which are combinations of two different nanoparticles) and tri-hybrid nanofluids (which are the combination of three different nanoparticles) [6]. The analysis of entropy is an important aspect of fluid flow studies, which is used to describe the impact of different physical parameters of the model. Nanofluid flow under the influence of the entropy regime was analyzed by Shafee et al. [7] Elnaqeeb et al. [8], Mohammed et al. [9], and Animasaun et al. [10]. They all focused on the analysis of heat transfer within the existence of entropy.

An important kind of convection in terms of temperature difference, Marangoni convection, has been introduced by researchers for thermal device applications. Some authors [11,12] analyzed the effects of radiation on nanofluid flux in the presence of Marangoni convection. Golia and Viviani [13] investigated the influence of Marangoni convection on nonisoproduct flow. Chamkha et al. [14] considered combined Marangoni and mixed convection in fluid flows on a free surface. Others [15,16] investigated the effects of radiation effects on Marangoni convection over a plane surface. Hayat et al. [17] considered Marangoni convection in a case of nonlinear stretching. Convection in various geometries in the case of nanofluids applied for heat transfer was studied by Ali et al. [18] and Kumar et al. [19].

Entropy generation must be analyzed in the case of nanofluids, but studies in this field are scarce in the literature. Akbarzadeh et al. [20], Shezad et al. [21], and Nasir et al. [22] concentrated on irreversibility analysis in various geometries considering nanofluids.

The homotopy analysis method (HAM) was used by Shijun [23] to solve nonlinear problems. Nasir et al. [24], Usman et al. [25], Saeed et al. [26], and Bilal et al. [27] used this method for highly nonlinear and complex problems. Researchers have also validated this method by comparing this method with other numerical methods.

In this study, the flow dynamics framework of a Marangoni tri-hybrid nanofluid flow was analyzed for thermal applications. Water was used as the common liquid containing nanomaterials such as MWCNTs, MoS_2_, and SiO_2_. MWCNT/H_2_O (nanofluid), MWCNT+ MoS_2_/H_2_O (hybrid nanofluid), and MWCNT + MoS_2_ + SiO_2_/H_2_O (tri-hybrid nanofluid) were all studied with this model. In addition to the aforementioned goals, the following topics were explored:

➢ MWCNT + MoS_2_ + SiO_2_/H_2_O (tri-hybrid nanofluid) flow for thermal applications;
➢ Irreversibility analysis under the influence of the modeled parameters;
➢ Pattern of fluid motion under a magnetic field and thermal radiation;
➢ Marangoni convection was considered in flow analysis;
➢ The obtained system was solved using the HAM approach.

## 2. Formulation

The steady flow of tri-hybrid nanofluids containing MoS_2_, SiO_2_, and MWCNTs is used in a single base fluid of H_2_O to produce the tri-hybrid nanofluid. Marangoni convection is considered in terms of the temperature difference between the free surface and liquid. A magnetic field has practical implications for the motion of a liquid in a slanted position. Thermal radiation is also imposed to strengthen the heat transfer analysis. The basic flow equations are as follows:

(1) $$\frac{\partial \;u}{\partial \;x}+\frac{\partial \;v}{\partial \;y}=0,$$

(2) $$u\frac{\partial u}{\partial x}+v\frac{\partial u}{\partial y}=\upsilon_{tnf}\frac{\partial^{2}u}{\partial y^{2}}-⟮\frac{\sigma_{thnf}B_{0}^{2}}{\rho_{thnf}}⟯u\;\sin^{2}(\Omega ),$$

(3) $$u\frac{\partial T}{\partial x}+v\frac{\partial T}{\partial y}=\frac{k_{thnf}}{{\left(\rho c_{p}\right)}_{thnf}}⟮\frac{\partial^{2}T}{\partial y^{2}}⟯-\frac{1}{{\left(\rho c_{p}\right)}_{thnf}}⟮\frac{\partial q}{\partial y}⟯.$$

The physical conditions are adjusted as follows:

(4) $$\begin{array}{l} \frac{\mu_{thnf}}{\mu_{f}}\frac{\partial u}{\partial y}=-\frac{d\sigma }{dT}\frac{\partial T}{\partial x},v\;=\;0,\;T-T_{w}=0,\;\;\;\mathrm{at}\;\;\;\;y\to 0,\\ u=0,\;T-T_{\infty }=0\;\;\;\;\mathrm{at}\;\;\;\;y\to \infty . \end{array}$$

Similarity variants are used to transform the above equations into [17]:

(5) $$\left[\eta ,\psi ,\Theta ,A,C_{1},C_{2},u,v\right]=\left[\begin{array}{l} yC_{2}x^{\frac{\left(r-1\right)}{3}},C_{1}x^{\frac{\left(2+r\right)}{3}}f(\eta ),\;\frac{T-T_{\infty }}{Ax^{\left(1+r\right)}},\frac{\Delta T}{L^{\left(1+r\right)}},\\ {⟮\frac{\sigma_{T}A\mu_{f}}{\rho_{f}^{2}}⟯}^{\frac{1}{3}},{⟮\frac{\sigma_{T}A\rho_{f}}{\mu_{f}^{2}}⟯}^{\frac{1}{3}},\frac{\partial \psi }{\partial y},-\;\frac{\partial \psi }{\partial x}\; \end{array}\right].$$

where components *u* and *v* are used to represent the velocity in two-dimensional space; *T* is the temperature and *q* is thermal radiation, respectively. Using the concepts of Rosseland’s approximation $q=-\frac{4}{3}\frac{\sigma^{*}}{k^{*}}\frac{\partial T}{\partial y}$, Equation (4) shows the physical conditions with the thermal concept for Marangoni convection. Equation (5) is used to transform the PDEs into ODEs. Equation (5) transforms all the main Equations (1)–(4) into a simplified form.

(6) $$f^{\prime \prime \prime }+\frac{\rho_{thnf}}{\rho_{f}}\frac{\mu_{f}}{\mu_{thnf}}\left[\frac{2+r}{3}ff^{\prime \prime }-\frac{1+2r}{3}{\left(f^{\prime }\right)}^{2}\right]-\frac{\mu_{f}}{\mu_{thnf}}\frac{\sigma_{thnf}}{\sigma_{f}}M\sin^{2}(\Omega )f^{\prime }=0,$$

(7) $$⟮\frac{k_{thnf}}{k_{f}}+\frac{4}{3}Rd⟯\Theta^{\prime \prime }+\mathrm{Pr}\frac{{\left(\rho cp\right)}_{thnf}}{{\left(\rho cp\right)}_{f}}\left[\frac{2+r}{3}f\Theta^{\prime }-\left(1+r\right)\Theta f^{\prime }\right]=0,$$

The transformed physical conditions from (6) and (7) are distorted as:

(8) $$f(0)=0,\;\frac{\mu_{thnf}}{\mu_{f}}f^{\prime \prime }(0)=-1,\;\;\;f^{\prime }(\infty )=\;\Theta (\infty )=0,\Theta (0)=1.$$

The following are the basic models of the thermophysical characteristics of ternary hybrid nanofluids:

(9) $$\frac{\mu_{thnf}}{\mu_{f}}=\frac{1}{{\left(1-\phi_{1}\right)}^{2.5}{\left(1-\phi_{2}\right)}^{2.5}{\left(1-\phi_{3}\right)}^{2.5}},\frac{\rho_{thnf}}{\rho_{f}}=\left(1-\phi_{1}\right)\left[\left(1-\phi_{2}\right)\left\{\left(1-\phi_{3}\right)+\phi_{3}\frac{\rho_{3}}{\rho_{f}}\right\}+\phi_{2}\frac{\rho_{2}}{\rho_{f}}\right]+\phi_{1}\frac{\rho_{1}}{\rho_{f}},$$

(10) $$\frac{k_{thnf}}{k_{hnf}}=⟮\frac{k_{1}+2k_{hnf}-2\phi_{1}\left(k_{hnf}-k_{1}\right)}{k_{1}+2k_{hnf}+\phi_{1}\left(k_{hnf}-k_{1}\right)}⟯,\frac{k_{hnf}}{k_{nf}}=⟮\frac{k_{2}+2k_{nf}-2\phi_{2}\left(k_{nf}-k_{2}\right)}{k_{2}+2k_{nf}+\phi_{2}\left(k_{nf}-k_{2}\right)}⟯,\;\frac{k_{nf}}{k_{f}}=⟮\frac{k_{3}+2k_{f}-2\phi_{3}\left(k_{f}-k_{3}\right)}{k_{3}+2k_{f}+\phi_{3}\left(k_{f}-k_{3}\right)}⟯,\frac{\sigma_{thnf}}{\sigma_{hnf}}=\frac{\left(1+2\phi_{1}\right)\sigma_{1}+\left(1-2\phi_{1}\right)\sigma_{hnf}}{\left(1-\phi_{1}\right)\sigma_{1}+\left(1+\phi_{1}\right)\sigma_{hnf}}.$$

(11) $$\frac{\sigma_{hnf}}{\sigma_{nf}}=\frac{\left(1+2\phi_{2}\right)\sigma_{2}+\left(1-2\phi_{2}\right)\sigma_{nf}}{\left(1-\phi_{2}\right)\sigma_{2}+\left(1+\phi_{2}\right)\sigma_{nf}},\frac{\sigma_{nf}}{\sigma_{f}}=\frac{\left(1+2\phi_{3}\right)\sigma_{3}+\left(1-2\phi_{3}\right)\sigma_{f}}{\left(1-\phi_{3}\right)\sigma_{3}+\left(1+\phi_{3}\right)\sigma_{f}}.$$

(12) $$\frac{{\left(\rho cp\right)}_{thnf}}{{\left(\rho cp\right)}_{f}}=\left(1-\phi_{1}\right)\left[\left(1-\phi_{2}\right)\left\{\left(1-\phi_{3}\right)+\phi_{3}\frac{{\left(\rho cp\right)}_{3}}{{\left(\rho cp\right)}_{f}}\right\}+\phi_{2}\frac{{\left(\rho cp\right)}_{2}}{{\left(\rho cp\right)}_{f}}\right]+\phi_{1}\frac{{\left(\rho cp\right)}_{1}}{{\left(\rho cp\right)}_{f}},$$

The heat transport is mathematically expressed as [17]:

(13) $$Nu_{x}=\frac{xq_{w}}{k_{f}\left(T_{w}-T_{0}\right)},q_{w}=-\left[k_{thnf}+\frac{16\sigma^{*}T_{\infty }^{3}}{3k^{*}}\right]{⟮\frac{\partial T}{\partial y}⟯}_{y=0},$$

The non-dimensional form of $Nu_{x}$ is

(14) $$Nu_{x}=-C_{2}x^{\frac{\left(2+r\right)}{3}}⟮\frac{k_{thnf}}{k_{f}}+\frac{4}{3}Rd⟯\Theta^{\prime }\left(0\right).$$

## 3. Entropy Rate

Entropy is a fundamental idea in mathematics and the physical theories. Entropy is vital to continuum mechanics, thermodynamics, environmental science, and finance [21,22,23,24]. Entropy is a physical idea that is conditional on the second law of thermodynamics, and entropy increases in an insulated structure as a result of any activity. The idea relevant to the model problem is stated as:

(15) $$S_{g}=\frac{1}{T_{\infty }^{2}}⟮k_{hnf}+\frac{16\sigma^{*}T_{h}^{3}}{3k^{*}}⟯{⟮\frac{\partial T}{\partial y}⟯}^{2}+\frac{\mu_{hnf}}{T_{\infty }}{⟮\frac{\partial u}{\partial y}⟯}^{2}+\frac{\sigma_{hnf}B_{0}^{2}}{T_{\infty }}u,$$

(16) $$S_{G}=⟮\frac{k_{hnf}}{k_{f}}+Rd⟯\lambda {\left(\Theta^{\prime }\right)}^{2}+Br\left[{\left(f^{\prime \prime }\right)}^{2}{\left(1-\phi_{1}\right)}^{-2.5}{\left(1-\phi_{2}\right)}^{-2.5}+M{\left(f^{\prime }\right)}^{2}\right].$$

where $\lambda =\frac{T_{w}-T_{\infty }}{T_{\infty }},S_{G}=\frac{S_{g}T_{\infty }}{\left(T_{w}-T_{\infty }\right)}$ are the differences in temperatures and entropy rate, respectively.

### Bejan Number

The ratio of the heat transfer that occurs due to irreversibility and the whole irreversibility is called the Bejan number.

(17) $$Be=\frac{\frac{k_{hnf}}{k_{f}}{\left(\Theta^{\prime }\right)}^{2}\lambda }{\lambda ⟮Rd+\frac{k_{hnf}}{k_{f}}⟯{\left(\Theta^{\prime }\right)}^{2}+Br\left[{\left(f^{\prime \prime }\right)}^{2}{\left(1-\phi_{1}\right)}^{-2.5}{\left(1-\phi_{2}\right)}^{-2.5}+Mf^{\prime }\right]}.$$

## 4. Results and Discussion

**Figure 1:**
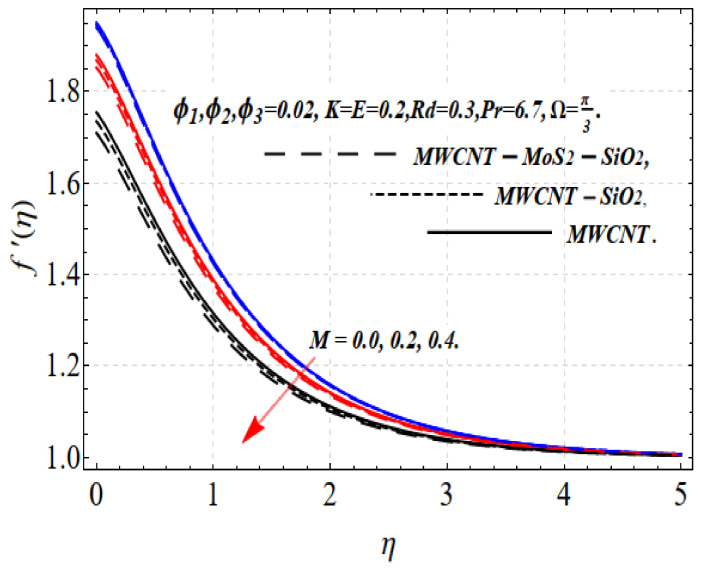
$f^{\prime }(\eta )$ via M.

**Figure 2:**
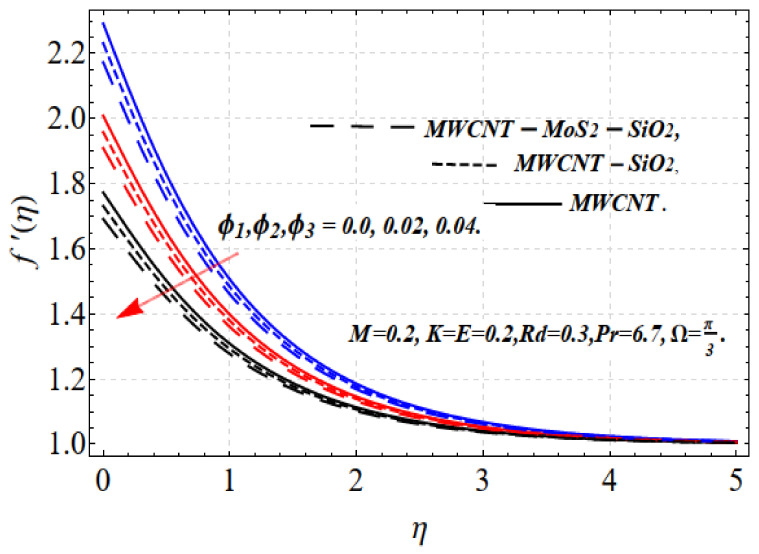
$f^{\prime }(\eta )$ vs. $\phi_{1},\phi_{2},\phi_{3}$.

**Figure 3:**
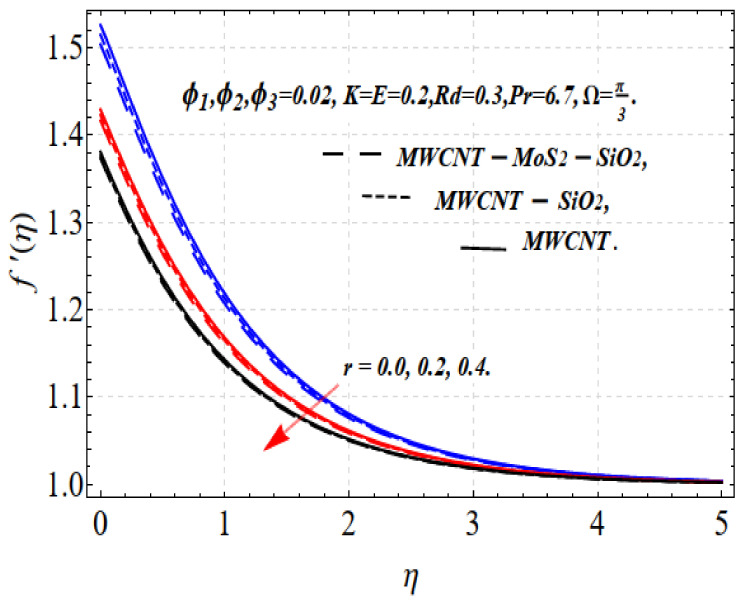
$f^{\prime }(\eta )$ vs. r.

**Figure 4:**
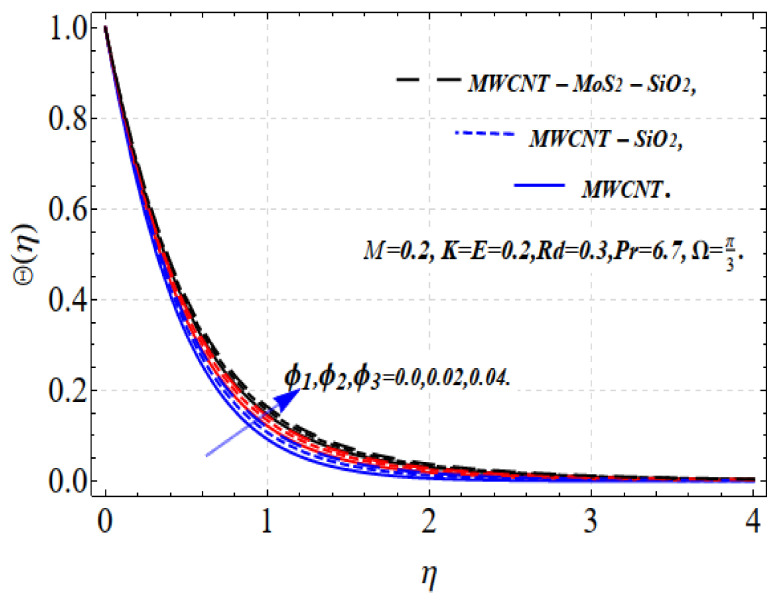
Θ(η) vs. $\phi_{1},\phi_{2},\phi_{3}$.

**Figure 5:**
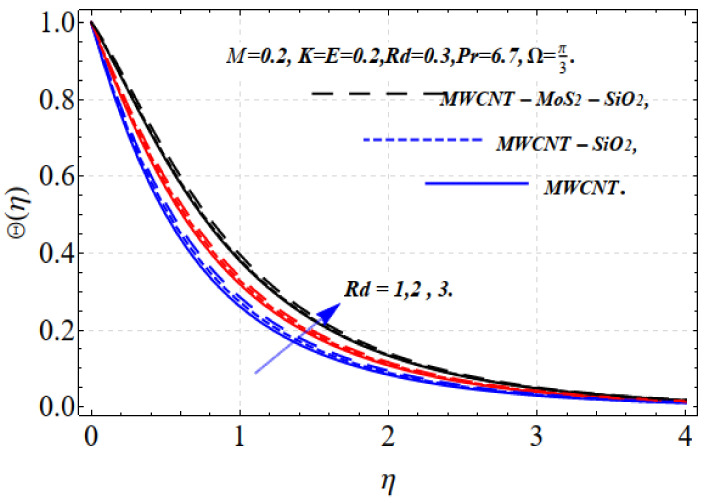
Θ(η) vs. Rd.

The solution to the proposed model was obtained using the HAM technique. The outputs are displayed in Figure 1, Figure 2, Figure 3, Figure 4 and Figure 5 and numerically in Table 1.

Figure 1 shows the relationship of velocity $f^{\prime }(\eta )$ with the magnetic field *M*. A larger *M* increases the resistance force. The resistance of a fluid is represented by the Lorentz force, which can oppose the fluid movement and is the source of the magnetic field composition. The fluid $f^{\prime }(\eta )$ profile declines with illumination, as shown in Figure 1. As a result, the boundary surface thickness gradually decreases as *M* increases. Additionally, when *M* = 0, the maximum $f^{\prime }(\eta )$ occurs within the computational region. The resistance slows the fluid motion.

The velocity of an electrically conducting nanofluid produces Lorentz force, which is associated with deceleration. For this reason, $f^{\prime }(\eta )$ displays a contradictory tendency, where the strength of the three types of nanoparticles increases with increasing magnitude. Consequently, the velocity of the nanofluids also decreases. In comparison with nano and hybrid nanosuspensions, tri-hybrid nanosuspensions have a smaller amplitude $f^{\prime }(\eta )$.

The influence of $\phi_{1},\phi_{2},\phi_{3}\;\mathrm{and}\;r$ is shown in Figure 2 and Figure 3. A larger volume fraction and higher nonlinearity decrease the movement of the fluid because of the resistive force. Accordingly, the motion $f^{\prime }(\eta )$ decreases. Physically, this occurs because higher values of $\phi_{1},\phi_{2},\phi_{3}$ result in more nanoparticles in the base fluid, which increases the resistance of the related fluid and decreases the velocity of the fluid. This indicates that different nanofluid base liquids are advantageous depending on the specific requirements of industry and other applications. The increase in the size of the solid volume fraction causes the viscosity of the fluid to increase and oppose the nanofluid stream under the influence of shear stress. Consequently, the speed of the nanofluid decreases. Tri-hybrid nanoparticles are more efficient than hybrid nanoparticles, liquids, and nanofluids in terms of the smallest quantity required for the velocity field.

Figure 4 and Figure 5 depict the thermal distribution Θ(η) against $\phi_{1},\phi_{2},\phi_{3}\;\mathrm{and}\;Rd$. An increase in $\phi_{1},\phi_{2},\phi_{3}\;\mathrm{and}\;Rd$ .increases the thermal efficiency (conductivity) in the thermal system, which increases the temperature Θ(η). The influence on all the obtained results is comparatively strong when using the tri-hybrid nanofluids. For MWCNTs-MoO_2_-SiO_2_, MWCNTs-MoO_2_, and MWCNTs nanofluids, the incorporation of $\phi_{1},\phi_{2},\phi_{3}$ substantially impacts the considered thermophysical aspects. Compared with MWCNTs-MoO_2_-SiO_2_, the $k_{nf}$, and $\mu_{nf}$ of the nanofluid markedly improve. For the dynamics of the nanoparticle volume fraction for various nanofluids, the dual feature can be explained. The fluid cooling effect of MWCNTs-MoO_2_-SiO_2_ nanofluid is strengthened as the value of $\phi_{1},\phi_{2},\phi_{3}.$ increases. The influence of $\phi_{1},\phi_{2},\phi_{3}\;\mathrm{and}\;r$ is shown in Figure 2 and Figure 3. A higher volume fraction and nonlinearity decrease the movement of the fluid because of the resistive force. Accordingly, the $f^{\prime }(\eta )$ motion decrease. Physically, this occurs because a higher value of $\phi_{1},\phi_{2},\phi_{3}$ results in more nanoparticles in the base fluid, which increases the resistance of the related fluid and slows the velocity of the fluid. This indicates that the use of a certain nanofluid base liquid is advantageous depending on the application requirements. The increase in the size of the solid volume fraction causes the viscosity of the fluid to increase and oppose the nanofluid stream under the influence of shear stress. Consequently, the speed of the nanofluid decreases. Tri-hybrid nanoparticles are more efficient than hybrid nanoparticles, liquids, and nanofluids in terms of the smallest quantity being needed for the velocity field. Improvements in the temperature distribution and the associated thickness of the boundary film result from the increase in the Rd estimate. An improvement in the surface heat stream is induced by Rd. This leads to an improvement in the temperature in the boundary layer area. The tri-hybrid nanoparticles, in this case, are superior compared with hybrids and nanocomposites.

**Figure 6:**
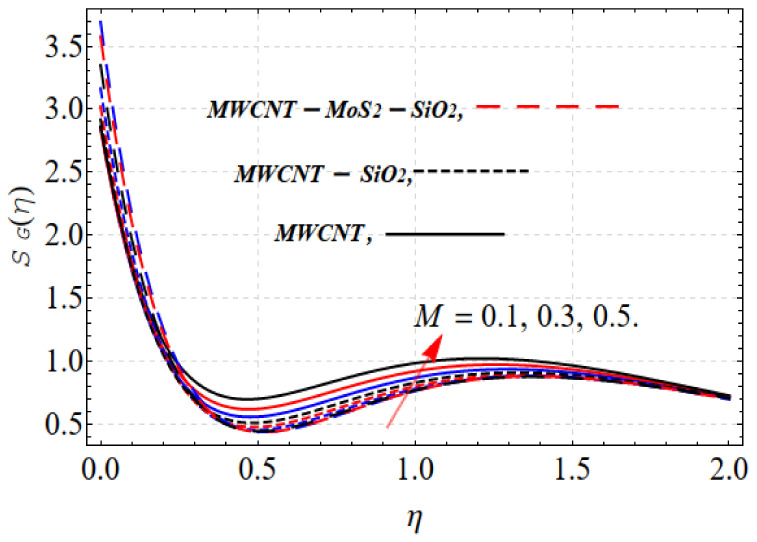
SG(η) vs. M.

**Figure 7:**
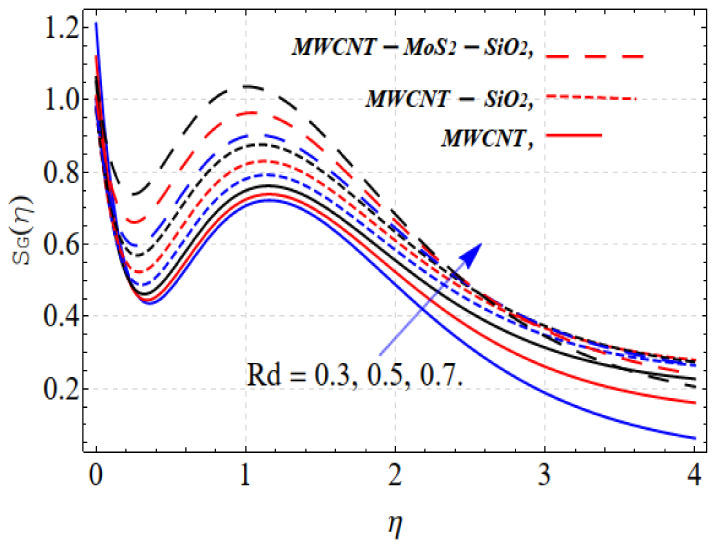
SG(η) vs. Rd.

The entropy regimen increases due to the increase in *M*. The Lorentz force leads to disturbances and, consequently, the heat in the thermal system increases, as shown in Figure 6. The opposite trend occurs with the Bejan number, as revealed in Figure 7.

**Figure 8:**
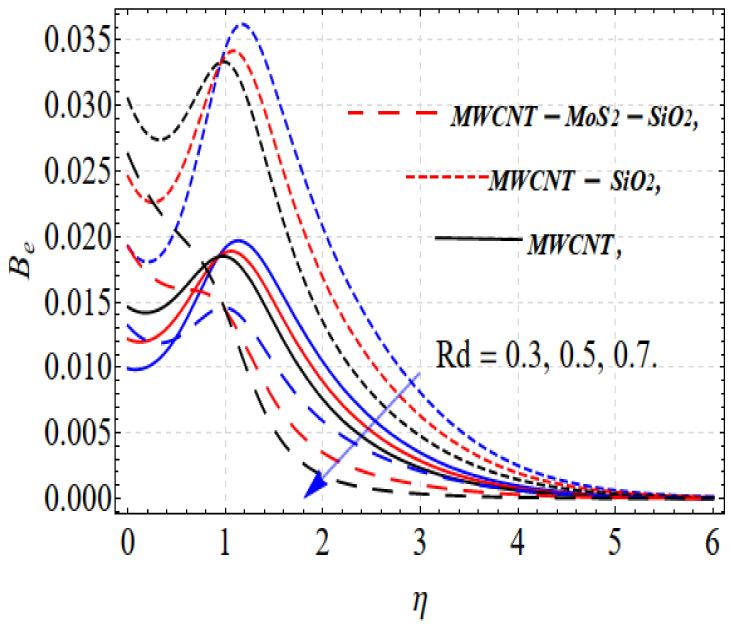
Be vs. Rd.

Similar results are obtained: increasing temperature radiation increases entropy generation, as shown in Figure 8.

Table 1 shows the percent progress in heat transfer rate attributable to the estimation of the volumetric fraction of nanoparticles. The results show that hybrid nanofluids are more capable of improving the thermal efficiency of conventional fluids.

**Figure 9:**
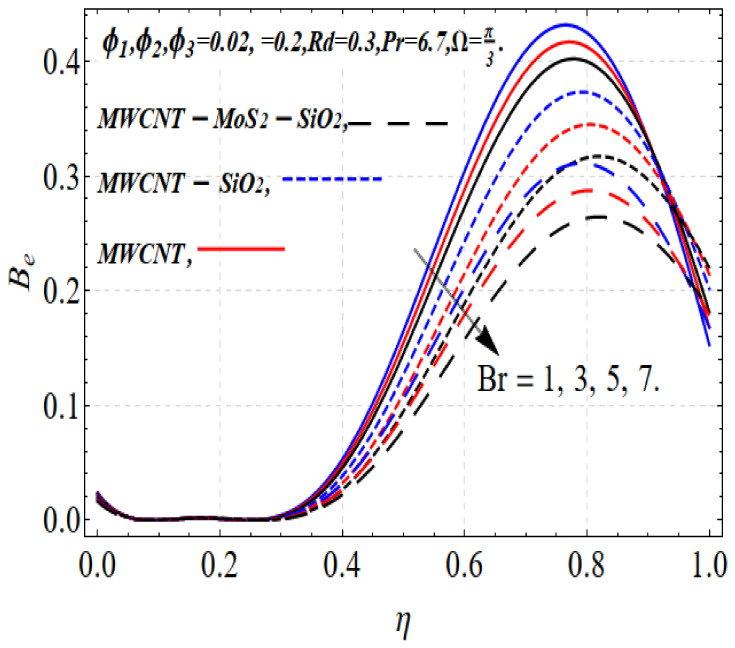
Be vs. Br.

**Figure 10:**
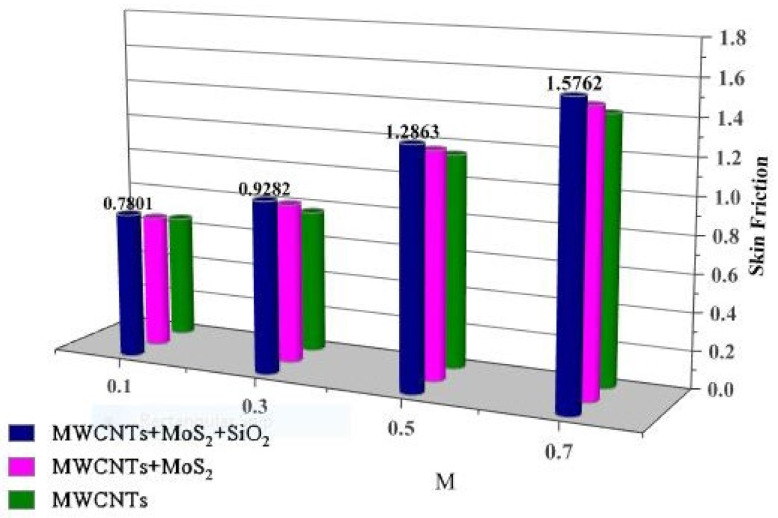
Drag force vs. M.

The effects of the Bejan number (Be) on the Brinkman number (Br) are shown in Figure 9. Br decreases with the increase in Br. The thermal field increases with increasing Brinkman number, but the opposite occurs with the Bejan number. The resistance force increases with increasing magnetic field parameter values, which consequently increases the surface friction. This effect is more precise in the case of tri-hybrid nanofluids, as shown in Figure 10.

**Figure 11:**
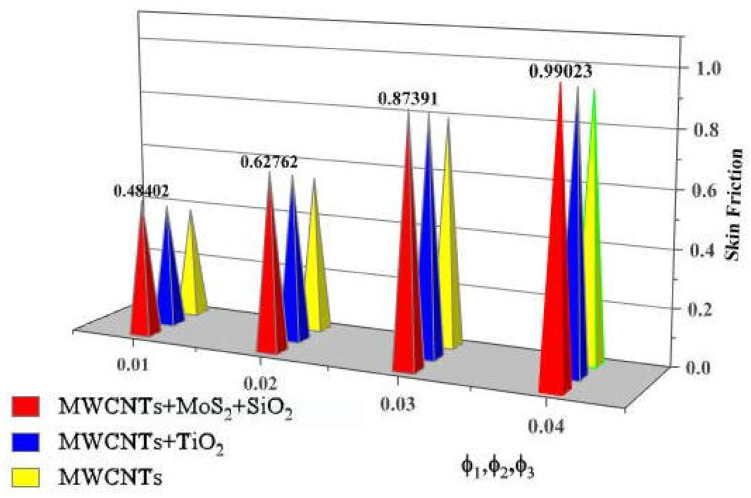
Drag force vs. $\phi_{1},\phi_{2},\phi_{3}$.

Larger volume fractions of solid nanoparticles increase the resistance force and create obstacles during fluid movement, which increases skin friction, as shown in Figure 11.

**Figure 12:**
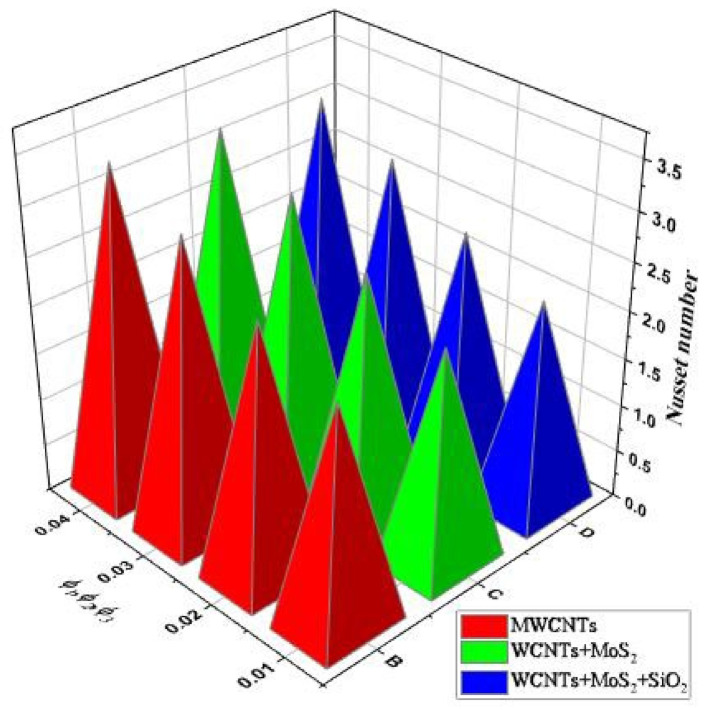
Nusselt number vs. $\phi_{1},\phi_{2},\phi_{3}$.

The thermal profile increases as the volume fraction of the nanoparticles grows, which is comparatively larger using tri-hybrid and hybrid nanofluids, as displayed in Figure 12. Hence, the heat transfer rate increases with the increase in the nanoparticle volume fraction and is more prominent for tri-hybrid nanofluids.

**Figure 13:**
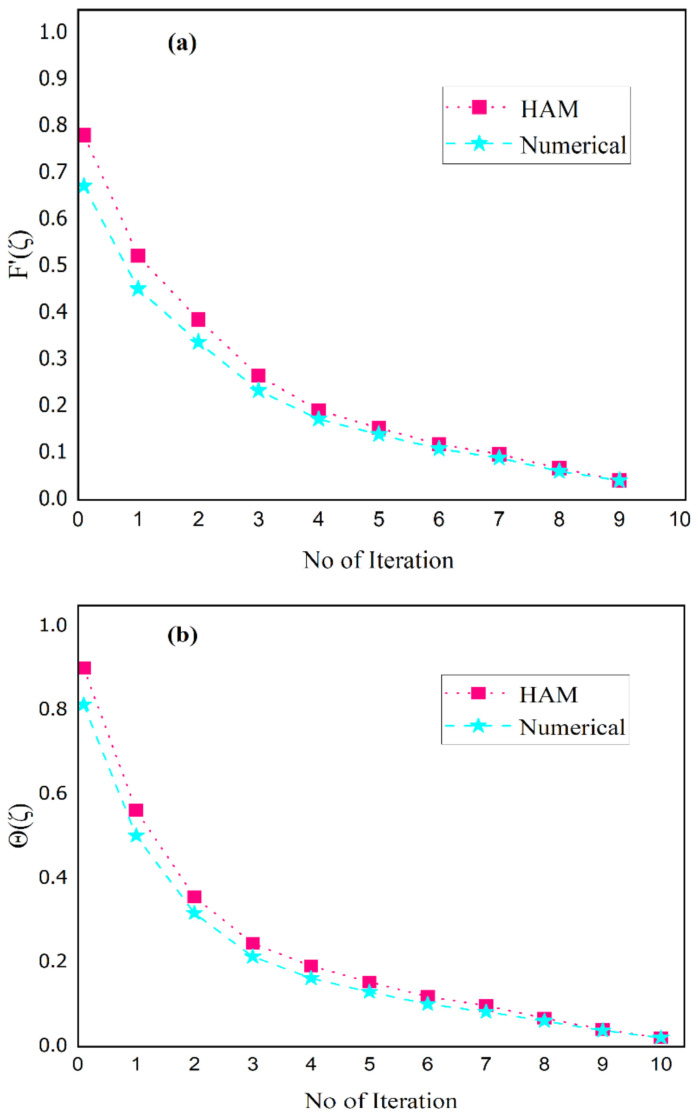
Comparison of HAM and numerical solution subject to (**a**) $f^{\prime }\left(\eta \right)$ and (**b**) $\Theta \left(\eta \right)$ by taking ϕ=0 (H_2_O case).

Figure 13a,b presents a validation of the HAM technique with a numerical (shooting) scheme; a strong correlation is shown for $f^{\prime }(\eta )$ with Θ(η).

## 5. Conclusions

Researchers optimize the properties of single-particle nanofluids by varying the volumetric fraction of nanoparticles. However, there is a limit to this because of the question of the compromise in the net negative of the increase in viscosity. To avoid this conflict and provide better heat transfer, researchers introduced hybrid nanofluids, followed by tri-hybrid nanofluids. The present results show that tri-hybrid nanofluids are more efficient at improving the heat transfer of traditional fluids.

The results of this analysis are as follows: According to the results of a comparative study, when using an MWCNTs-MoO_2_-SiO_2_ nanofluid, the physical restrictions have a relatively substantial effect because MWCNTs-MoO_2_-SiO_2_ has powerful thermo-physical characteristics. In comparison with MWCNTs-MoO_2_-SiO_2_, the $k_{nf}$ and $\mu_{nf}$ of the nanofluid are significantly improved. In terms of the dynamics of nanoparticle volume fraction for various nanofluids, two features can be explained:

➢ Velocity is a disintegration function of the nonlinear term and the volume fraction of nanoparticles.
➢ The drag force increases with high values of M, $\phi_{1},\phi_{2},\phi_{3}$.
➢ For M and $\phi_{1},\phi_{2},\phi_{3}$, the heat flux improves.
➢ Intensifying the magnetic variable lowers the velocity; the trend is more pronounced in terms of tri-hybrid nanofluids.
➢ Increasing the Brinkman number decreases the Bejan number.
➢ A strong magnetic field increases the generation of entropy.
➢ The thermal distribution is the same for the thermal field and volumetric fraction of nanoparticles.
➢ The stable dispersion of the 1% nanoparticle volume fraction increases heat transfer by 3.71%, 4.32%, and 5.41% in the case of the nanofluid, the hybrid nanofluid, and the tri-hybrid nanofluid, respectively. In addition, in the case of 2% and 3% volume fractions, the ratio of heat transfer increases in a similar fashion. This percentage increase in heat transfer confirms that tri-hybrid nanofluids are more suitable for improving the thermal conductivity of base fluids.

## Figures and Tables

**Table 1:** Heat transfer calculated as a percentage for each nanoparticle.

$\phi_{1},\phi_{2},\phi_{3}$	$Nu_{x}\;\mathrm{MWCNTs}$	$Nu_{x}\;\mathrm{MWCNTs}+{\mathrm{MoS}}_{2}$	$Nu_{x}\;\mathrm{MWCNTs}+{\mathrm{MoS}}_{2}+{\mathrm{SiO}}_{2}$
**0.00**	2.9057 0% Increase	2.9057 0% Increase	2.9057 0% Increase
**0.01**	3.0135 3.71% Increase	3.0135 4.32% Increase	3.0135 5.41% Increase
**0.02**	3.0763 5.87% Increase	3.0763 6.62% Increase	3.0763 10.37% Increase
**0.03**	3.2332 11.27% Increase	3.2332 13.46% Increase	3.2332 17.62% Increase

## Data Availability

All the research data exist in the article.

## Nomenclature

**List of Symbols**	**Greek Symbols**
u, v Velocities components $\left({\mathrm{ms}}^{-1}\right).$	$\mu_{nf}$ Dynamic nanofluid viscosity $\left(\mathrm{mPa}\right).$
$B_{0}$ Magnetic field strength $\left({\mathrm{NmA}}^{-1}\right).$	η Similarity variable
$C_{f}$ Skin friction coefficient	$\rho_{nf}$ Nanofluid density $\left({\mathrm{Kgm}}^{-3}\right).$
T Fluid temperature $\left(K\right).$	$\rho_{f}$ Base fluid density $\left({\mathrm{Kgm}}^{-3}\right).$
${\left(C_{p}\right)}_{f}$ Specific heat of the base fluid $\left(J/\mathrm{kgK}\right).$	$\sigma_{nf}$ Base fluid electrical conductivity (Sm^−1^)
$T_{\infty }$ Free surface temperature $\left(K\right).$	$\mu_{f}$ Dynamic viscosity of the base fluid $\left(\mathrm{mPa}\right).$
M Magnetic parameter	Rd Thermal radiation parameter
f Dimensional velocity profiles	$k_{f}$ Thermal conductivity of base fluid (Wm K^−1^)
$T_{w}$ Wall surface temperature $\left(K\right).$	**Subscripts**
Pr Prandtl number	*Thnf* Tri-hybrid nanofluid
$\sigma_{nf}$ Electrical conductivity of nanofluid (Sm^−1^)	nf Nanofluid
Νu Nusselt number	f Base fluid
Θ Dimensional heat profiles	**Abbreviations**
ϕ Volume fraction of nanoparticles	HAM Homotopy analysis method
$k_{nf}$ Thermal conductivity $\left(Wm^{-1}K^{-1}\right)$	MHD Magneto-hydrodynamics
